# Visco-Elastic Honeycomb Structures with Increased Energy Absorption and Shape Recovery Performance Using Buckling Initiators

**DOI:** 10.3390/polym15163350

**Published:** 2023-08-09

**Authors:** Colleen M. Murray, Min Mao, Jungjin Park, John Howard, Norman M. Wereley

**Affiliations:** 1 Composites Research Laboratory, Department of Aerospace Engineering, University of Maryland, College Park, MD 20742, USA; cmmurray@umd.edu (C.M.M.); mmao@umd.edu (M.M.); pjj@umd.edu (J.P.); 2 Microsphere Material Solutions LLC, Rockville, MD 20852, USA; john@microspheresolutions.com

**Keywords:** honeycomb, out of plane compression, energy absorption, crashworthiness

## Abstract

Energy-absorbing materials have extensive applications in aerospace and automotive applications. Research has shown buckling initiators, or triggers, in energy-absorbing tubular structures increase the energy absorbed by encouraging the side panels to fold when loaded out of plane in compression conditions. Additively manufactured TPE honeycombs were designed in this study to include these buckling initiators, which introduced a slight decrease in initial weight, as well as initial stress concentrations, while improving crashworthiness characteristics. The samples with buckling initiators (1BI) showed an increase in crush efficiency when directly compared to their no buckling initiator (0BI) counterparts. The 1BI samples maintained an increased crush efficiency regardless of the strain rate used. The samples with 1BI were able to better equilibrate the peak stress with the plateau stress. These honeycomb samples were found to maintain their crush efficiency, even after multiple rounds of compression testing. The quasi-static 0BI samples experienced a 23.4% decrease in the peak stress after multiple rounds of compression testing, while the 1BI samples saw approximately a 23.0% decrease. The 1BI samples averaged a decrease in crush efficiency of 0.5%, while the 0BI samples saw a decrease in crush efficiency of 5%. As the strain rate increased, the crush efficiency for the 1BI samples showed an increase in performance, with a smaller degradation in crush efficiency over multiple uses. Visco-elastic honeycomb with buckling initiators has a higher energy absorption than samples with no buckling initiators when exposed to multiple impact cycles.

## 1. Introduction

Crashworthiness and energy-absorbing materials are of great interest to both automotive and aerospace industries due to the need for occupant protection. Work to date has shown that foam and honeycomb materials have a promising future in regard to energy absorption [1,2]. Using a controlled failure mechanism in a honeycomb will allow for a further increase in the energy absorption of the material.

Buckling initiators, sometimes referred to as trigger mechanisms, have been a commonly studied mechanism through which to introduce the controlled failure of a material structure. Two methods are typically used to introduce controlled failures [3]. For the purpose of this paper, they will be differentiated by the names buckling initiators and trigger mechanisms. Buckling initiators are voids introduced into the structure via a subtractive manufacturing method [4,5]. These initiators are purposeful stress concentrations intended to fail first, thus encouraging the structure to begin folding [6,7]. Traditionally, the part is completed before a drill is used to remove a portion of the material along the vertices. This method can be difficult to use due to the lack of control when implementing the buckling initiators. If the buckling initiator is implemented too high on the wall of the structure, there is the potential for the structure stiffness to decrease, causing the energy absorption to decrease [4]. Similarly, machining may introduce residual stresses into the part, potentially reducing the repeatability of the buckling performance.

On the other hand, an external trigger mechanism leads to controlled failures. These trigger mechanisms tend to be a plate placed at either the top or bottom of a tube [8,9,10]. These plates have a rectangular protuberance, so that when a compressive load is applied, the walls of the tube will experience a localized stress regime until failure occurs [11,12,13]. These plates, or trigger mechanisms, are easier to implement in a laboratory setting; however, they can be more difficult to implement in a large-scale setting. External trigger mechanisms also add weight to the structure; and so they are less desirable in vehicle applications.

Buckling initiators and trigger mechanisms have been extensively studied in tubular samples. There is extensive literature available regarding the behavior of tubes having different forms of defects. The most common cross-sections are circular and square tubes [14,15,16]. This concept has been studied in metals in the literature as far back as the 1960s, but the focus has been primarily on aluminum and its alloys [17,18,19,20,21].

Researchers saw the potential of combining lightweight aluminum with the high-strength, low-density design of the honeycomb [22,23,24,25]. Aluminum honeycomb is typically manufactured by gluing strips of aluminum together and inflating them to obtain the final structure [26,27,28]. The location of these strips of glue will increase the honeycomb wall thickness at those interfaces, causing the honeycomb to fail due to those interfaces as opposed to failing in buckling [29]. The thickness of the honeycomb walls will not be consistent using this manufacturing method. The varying wall thickness increases computational complexity, so consistent wall thickness has been assumed for simplicity [30,31]. Honeycomb can be designed to have various core shapes, including rectangular, triangular, and hexagonal [32,33,34,35,36,37,38]. Although aluminum is a common honeycomb material, honeycomb has been manufactured out of many materials, including—but not limited to—steel, fiber glass, paper, acrylonitrile butadiene sytrene (ABS), polylactic acid (PLA), and thermoplastic elastomer (TPE) [39,40,41,42,43,44].

Additively manufactured honeycombs like ABS, PLA, and TPE provide numerous benefits. The need for glue strips, unlike aluminum honeycomb, is eliminated. When oriented properly on the print bed, the fused filament fabrication (FFF) process will maintain the strength of the material, as many additive manufacturing studies have shown [45,46]. Additive manufacturing enables the precise fabrication of honeycomb geometry, especially with respect to a constant wall thickness. BIs can be introduced in any location and in any shape during printing so that residual stress from subtractive machining can be eliminated. Even with all of these benefits, additively manufactured honeycomb structures have not been extensively studied due to their brittle nature [47,48,49]. ABS and PLA exhibit brittle failure and high stiffness where it might be desirable to have buckling failure and low stiffness with the potential for shape recovery, which would suggest the use of a softer, visco-elastic material. Moreover, if a flexible, lighter weight visco-elastic material is identified, a honeycomb could be manufactured that could include buckling initiators and still be crashworthy [50].

Extensive research on honeycomb materials has been reported in the literature, including research on metallic honeycombs (ductile materials) and polymer material-based honeycombs (brittle materials). The original contributions of this current work are focused on: (1) the use of low shore hardness visco-elastic polymers with a goal of creating a low-pressure honeycomb that is suitable for occupant protection, (2) the introduction of buckling initiators (diamond-shaped holes) that are integrated into the structure to encourage the folding of the honeycomb faces and to improve the crush efficiency of the honeycomb structure, (3) to exploit the deformation recovery that may be possible when using buckling initiators of this type so that protection against multiple impacts can be provided (albeit with reduced effectiveness), and (4) to exploit additive manufacturing to fabricate these visco-elastic honeycomb structures with these buckling initiator features.

To achieve these goals, this study focuses on the influence of buckling initiators in a thermoplastic elastomer (TPE) honeycomb structure (or a visco-elastic honeycomb structure). The buckling initiators are expected to increase the crush efficiency due to the controlled buckling of the honeycomb faces (sides). Using an additive manufacturing process should minimize stress concentrations prior to testing, while ensuring that all walls are of the same thickness. Additive manufacturing enables the precise fabrication of cell size, as well as the location, shape, and size of the buckling initiators. The low shore hardness TPE should allow for the honeycomb to maximize the energy absorbed at low stroking loads as well. We will compare the energy absorption characteristics of visco-elastic honeycomb structures with buckling initiators located on the vertices (1BI samples) to those without buckling initiators (0BI samples) in order to assess their relative merits as crashworthy structures.

## 2. Materials and Methods

### 2.1. Manufacturing

**Figure 1:**
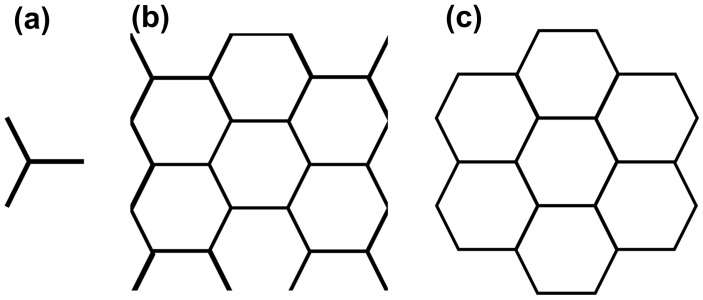
The unit cell of the tested honeycomb. (**a**) Accepted representative volume element according to Gibson; (**b**) originally tested sheet of honeycomb; and (**c**) Walls with a free edge obstructed the view of the honeycomb walls during crush, so that all such free edges were eliminated in the unit cell shown here, which was used in this study.

Honeycomb materials are commonly provided in sheets and have a repeating pattern. As explained in [27], the unit cell of a honeycomb structure may be represented as a vertex with its three adjoining walls (Figure 1a). Because of this lack of additional vertices, the interaction between cell BIs could not be easily observed in the experiment. As a result, preliminary tests were conducted on the samples that were square in shape; however, there were multiple free-standing walls (Figure 1b). When these honeycomb were tested, the walls collapsed in unrepeatable manners that were not representative of the honeycomb. These walls also blocked the view of the buckling initiators.

Because the goal of this study was to analyze the effects that BIs have on visco-elastic honeycomb, it was imperative that the BIs were visible. To maintain the representative performance of a full sheet of honeycomb and ensure that the BIs were visible, a representative volume element of six hexagonal cells surrounding a central hexagonal cell was selected (Figure 1c).

**Figure 2:**
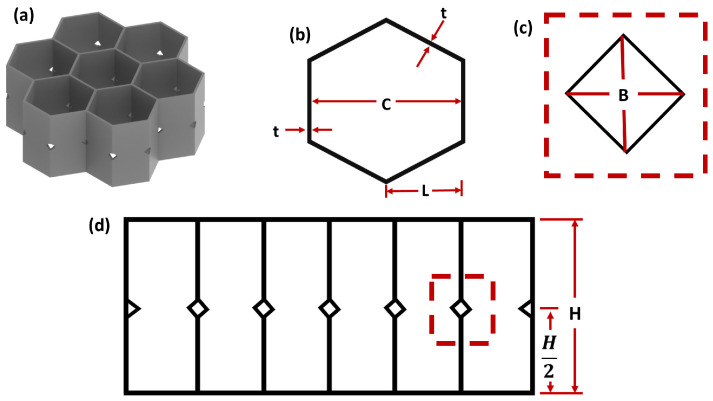
The dimensions used to manufacture the honeycomb. (**a**) The buckling initiators are voids on the vertices in the isometric view of the representative volume element. (**b**) The thickness and cell dimensions are shown in the top view. (**c**) Diamond-shaped buckling initiator. (**d**) The remaining dimensions in the side view. The honeycomb was designed so that *t* = 0.85 mm, *C* = 30 mm, *L* = 18.3 mm, and *H* = 30 mm. In addition, the honeycomb had diamond-shaped buckling initiators, where *B* = 4 mm.

Samples were manufactured using the Pro Series Flex TPE filament (MatterHackers, Lake Forest, CA, USA, 2018).The TPE had a Shore hardness of 98A. Samples were manufactured using a Diabase H-Series 3D Printer (Diabase Engineering, Longmont, CO, USA). This fused deposition modeling printer was selected for its proprietary extruders, which are compatible with flexible filaments with a Shore hardness ranging from 83D down to 60A. All samples had an overall height of 30 mm, with individual cells having a wall thickness of 0.85 mm, and an inscribed circle diameter of 30 mm (Figure 2). The samples with buckling initiators (1BI) had the BI half way up the wall at all vertices. The diamond shape was defined by a point-to-point interior dimension of 4 mm in each direction (Figure 2c). Honeycomb samples without buckling initiators (0BI) were also manufactured, for the purpose of comparison with the samples that had 1BIs, to assess how BIs affect performance.

### 2.2. Testing

All testing was conducted using an MTS-810 servo-hydraulic load frame (MTS, Eden Prairie, MN, USA). Three strain rates were studied to assess the effect of the strain rate on the buckling initiators. One group was tested under quasi-static compression conditions with a crush velocity, *V*, of 3 × $10^{-5}$ m/s or a strain rate of 0.001 s${}^{-1}$. The second group was tested at a velocity of 0.25 m/s (or a strain rate of 8.33 s${}^{-1}$), and the final group was tested at 0.5 m/s (or a strain rate of 16.67 s${}^{-1}$). Each group consisted of 0BI and 1BI samples that were compressed to 90% strain.

During testing, a camera recorded the loading and unloading of the honeycombs. For the quasi-static samples, a camera captured an image every 10 s, while the higher strain rates used a high-speed video camera (Redlake MotionProX3 Plus, San Diego, CA, USA) to record the behavior of the sample under compression loading. For all tests, the rebounding behavior was captured using a video camera as the sample was unloaded.

Each sample was compressed a total of three times with a 24 h waiting period between each crush experiment. The crush and strain recovery behavior of the honeycomb was studied using this data.

### 2.3. Performance Metrics

The analysis of these samples involves additional terminology. The stress was calculated by using the following:

(1) $$\sigma =\frac{F}{A_{uc}}$$

where *F* is the force and the area is calculated using the following:

(2) $$A_{uc}=\frac{21\sqrt{3}}{2}L^{2}$$

**Figure 3:**
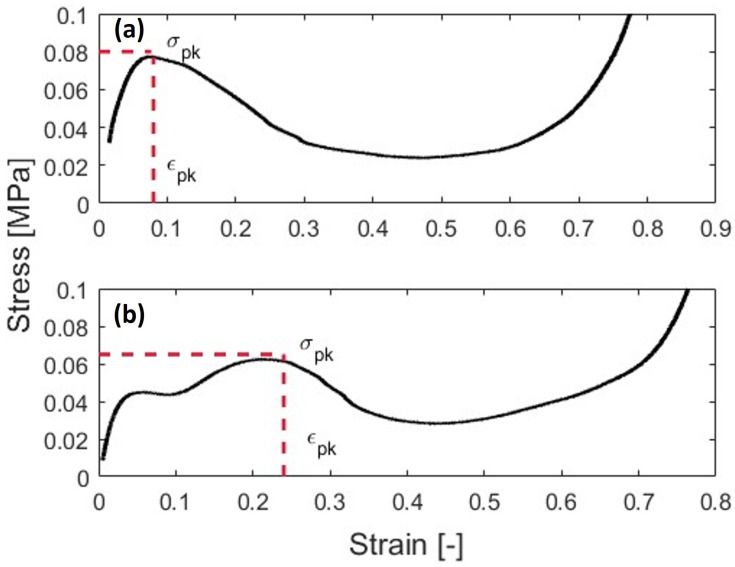
Honeycomb stress-strain curves of the out of plane honeycomb compression. (**a**) Representative curve for the samples with no buckling initiators. (**b**) The representative curve of the samples with a single symmetric buckling initiator. For each curve, the peak stress, $\sigma_{\mathrm{pk}}$, and its corresponding strain value, $\epsilon_{\mathrm{pk}}$, are identified.

Therefore, the stress is

(3) $$\sigma =\frac{F}{A_{uc}}=\frac{2F}{21\sqrt{3}L^{2}}$$

where *L* is the side length as shown in Figure 2d. At this point, the stress–strain curves can be computed from the test data for the sample analysis. As Figure 3 shows, there are two distinct behaviors for the samples with and without BIs. The methods for this will be described in order to characterize these curves.

To quantify strain energy density, the integral of the curve must be calculated:

(4) $$\begin{array}{ccc} EA & =\int_{0}^{\epsilon }\sigma \left(\epsilon \right)d\epsilon & {[J/m}^{3}]\; \end{array}$$

The strain energy density allows for additional metrics to be determined, showing the overall behavior of the materials. The first is the strain-dependent crush efficiency (SDCE), $\eta_{SD}$. The SDCE shows how crush efficient the material is at a given strain. It takes the strain energy density and divides it by a localized ideal strain energy density.

(5) $$\begin{array}{ccc} \eta_{SD} & =\frac{EA}{max[\sigma (0),\sigma (\epsilon )]\epsilon }=\frac{\sigma_{mc}}{max[\sigma (0),\sigma (\epsilon )]} & \; \end{array}$$

where $\sigma_{mc}$ is the mean crush stress. This value provides an average stress over the strain range. It is determined using the following:

(6) $$\begin{array}{ccc} \sigma_{mc} & =\frac{EA}{\epsilon } & \left[\mathrm{MPa}\right]\; \end{array}$$

If the efficiency is studied over the entire strain range, or ϵ=1, then the energy absorbed efficiency can be calculated as shown in the following equation:

(7) $$\begin{array}{ccc} \eta_{EA} & =\frac{EA}{max[\sigma (0),\sigma (\epsilon )]\epsilon }=\frac{\int_{0}^{\epsilon }\sigma \left(\epsilon \right)d\epsilon }{max[\sigma (0),\sigma (\epsilon )]} & \; \end{array}$$

This value will constantly increase until densification is achieved, at which point it will decline.

To complete this analysis, additional values must be identified. The first is the peak stress, which will be dependent on the stress calculation. The peak can be identified using the following:

(8) $$\begin{array}{ccc} \sigma_{pk} & =\frac{d\sigma }{d\epsilon }=0 & \left[\mathrm{MPa}\right]\; \end{array}$$

There are nominally three phases that are classically exhibited by an energy absorption material under crush. The first phase is the collapse. During this phase, the sample will deform but will present elastic deformation [51,52]. The second phase is a stress plateau, which is indicative of elastic buckling. During this phase, the sample absorbs energy transferred to the sample via cell wall collapse. This phase is called progressive collapse since the sample begins to fold. Densification occurs when voids in the honeycomb collapse and relative density and apparent stiffness greatly increase, so that the material is no longer usable as an energy absorbing material. Graphically, the stress will increase exponentially during this phase. The samples with and without BIs present these characteristics as shown in Figure 3.

## 3. Results

### 3.1. Buckling Features

**Figure 4:**
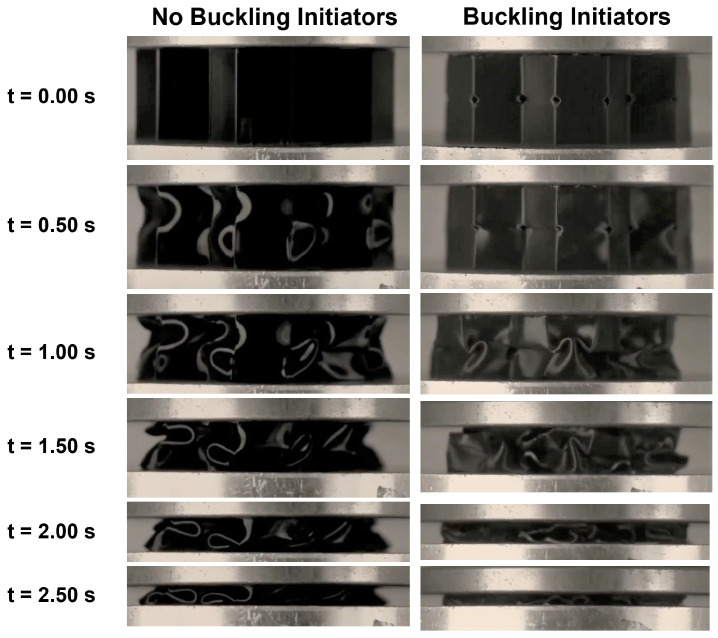
The process of crushing the honeycomb results with (**left**) no buckling initiators and (**right**) buckling initiators. This was recorded at a time interval of 0.50 s to analyze the folding mechanisms.

During crushing, samples without buckling initiators experience a repeatable folding pattern. Figure 4 shows the process of crushing the honeycomb with and without BIs. As the load is introduced to the sample, the honeycomb begins to form waves along its walls. As compression continues, the peaks of these waves become the hinge point for the folding to initiate. These samples showed a first buckling mode that is similar to the aluminum samples that Wilbert et al. recorded in their study [53]. The folds are symmetric around the middle of the wall until collapse occurs. During the collapse, some walls are pushed out, while others are pulled into the middle of the honeycomb, as depicted in Figure 4. This failure has been termed non-axisymmetric buckling [54].

The samples with BIs undergo two sub-phases during crushing in progressive collapse. The first phase is characterized by the BI closing, while the second phase is the formation of the hinge points. The BI controls the location of folding because it induces a stress concentration on the sample. As shown in the images taken at 0.50 s, the vertical corners of the initiator are pushed down so they come into contact, which pushes the horizontal corners further away, thus closing the BI. Once the BI is closed, the sample will attempt to collapse in a similar wave-type pattern. Because of the smaller wall height, this form of compression appears differently from the compression in the 0BI case, as shown in Figure 4. The collapse will instead form a series of organized folds due to the consistent locations of BIs, and this is called axisymmetric folding.

**Figure 5:**
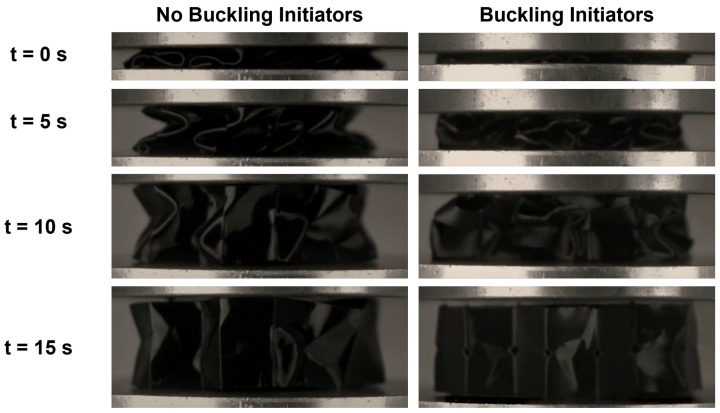
A sample with and without buckling initiators rebounding after an initial uni-axial compression test.

In a similar manner, the behavior of the samples was recorded as the load was removed. A constant crosshead velocity (≈2 mm/s) was used to unload the samples. The samples without buckling initiators showed the complex buckling modes as the platens moved apart. As the separation started, the folds opened wider, allowing for the walls to straighten more. Once the platens were entirely open, the sample was able to support itself, even with visible deformation on the walls, as shown in Figure 5.

When the load was removed from the samples with buckling initiators, the samples recovered from their strain by opening the hinges. The process of opening these folds required a significant amount of time. The buckling initiators opened last, allowing the samples to achieve over 90% of its original height. These samples took longer to rebound to their initial shape as shown in Figure 5, but were still able to withstand additional rounds of testing.

**Figure 6:**
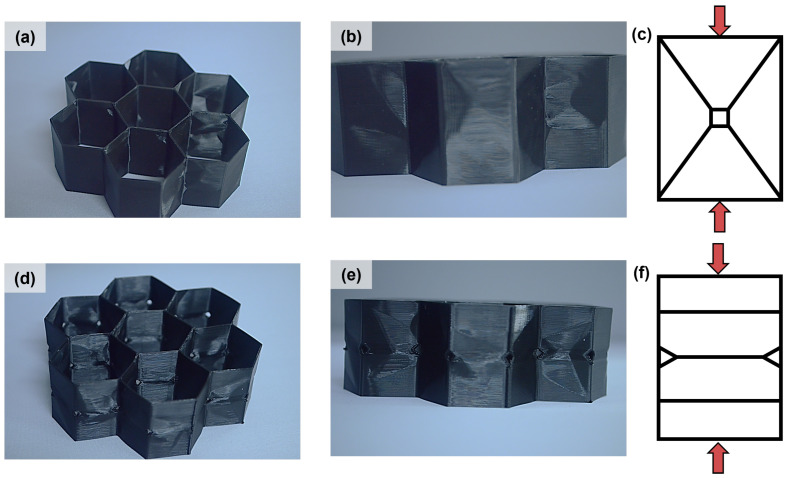
The failure mechanism is dependent on the presence of buckling initiators in the honeycomb. (**a**) The isometric view shows the shear buckling panels forming after 3 cycles of testing. (**b**) A front view of the triangular buckling panel. (**c**) Model of the shear buckling. (**d**) Isometric view shows folding failure after 3 cycles of testing. (**e**) The front view shows that the folding failure aligns with the neighboring cells. (**f**) Model of the folding failure.

Post analysis of the samples allows for the further behaviors to be recognized. After resting for 24 h after the 3rd cycle of compression, the samples exhibit unique folding patterns that were specific to their sample type. The samples without buckling initiators showed a distinct diamond pattern that formed on each cell wall, and these were characteristic of shear buckling. This process occurs when the stresses build up on the top and bottom of the cell until it becomes too large, forcing the wall to either protrude or recess into the cell. Figure 6 shows how a small rectangle on each of the walls will be undeformed due to the shear buckling mechanism, regardless of the number of times the sample is tested.

For the samples with BIs, a linear pattern became obvious after multiple rounds of testing. There were a series of horizontal pleats that formed on each face, and these corresponded to the collapse of the buckling initiators. With further observation, there were two other rows of pleats that were located halfway above the midpoint pleat and halfway below the midpoint pleat, as seen in Figure 6. These were the locations of the hinge points of the samples. The samples with buckling initiators favored a folding-based buckling failure due to the shortened wall height and the increased stress concentrations.

### 3.2. Quasi-Static Testing

**Figure 7:**
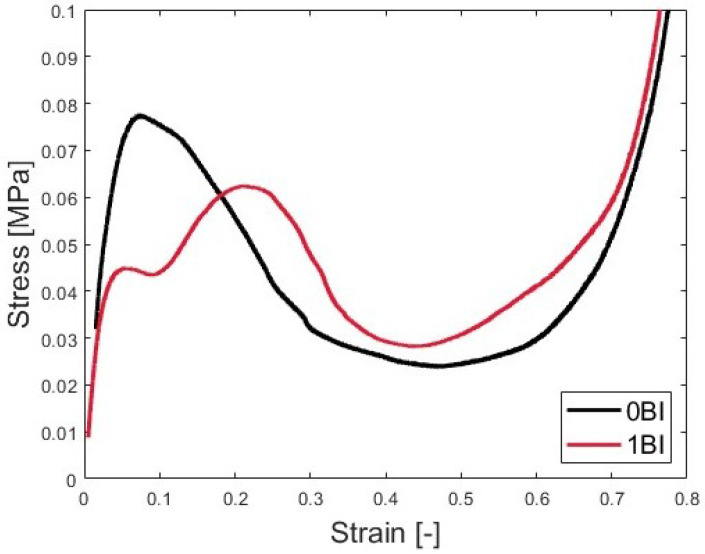
The stress- strain behavior of 0BI and 1BI samples.

Under quasi-static strain rates, performance of the honeycomb with and without buckling initiators were distinctly different. Looking at Figure 7, the samples with 0BI show a larger peak stress than the samples with 1BI. The 0BI sample has a peak stress of approximately 0.08 MPa, while the 1BI sample reaches approximately 0.064 MPa. The 1BI sample has three distinct regions where the stress increases. The first, where the sample experiences a 0 to 5% strain, corresponds to the initial loading of the sample. The second region of increase concludes at the peak stress. This region is dominated by the buckling initiators closing and generally lasts from approximately a 10 to 25% strain. The final region of increase corresponds to the densification of the sample. At this point, the sample has been compressed and is no longer useful.

The metrics from the stress–strain curve show significant differences between the two samples. The 0BI sample reached its peak stress at a corresponding strain of 0.07, while the samples with buckling initiators reached their peak stress at a strain of 0.21. The mean crush stress values also showed this deviation. The 0BI had a mean crush stress of 0.07 MPa, while the 1BI samples had a mean crush stress of 0.05 MPa. These peak stresses and strains can be identified graphically as well (Figure 7).

**Figure 8:**
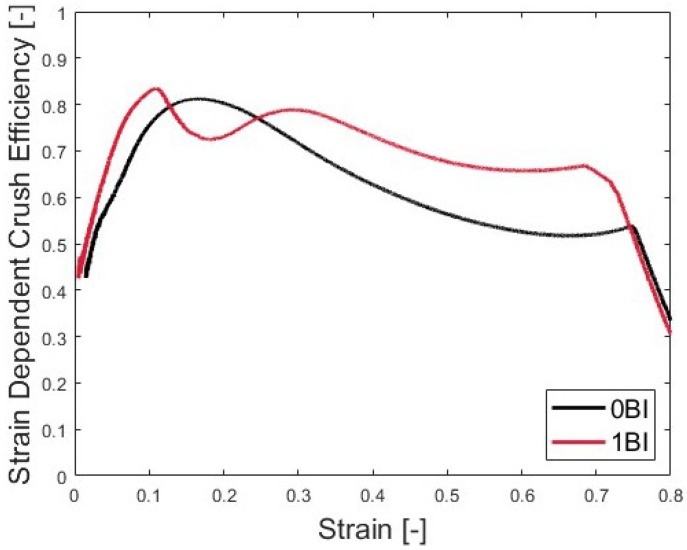
The strain dependent crush efficiency at quasi-static strain rates.

Using Equation (5), the strain-dependent crush efficiency shows the crush efficiency as a function of strain. As Figure 8 shows, the 0BI sample reached a maximum crush efficiency of 82% at a nominal strain of 0.15. After this point, the crush efficiency continued to decrease for the remaining usable strain range. This was a direct result of the high peak stress. When strain increased above the strain when the peak stress occurred, the stress continued to reduce dramatically below the peak stress (i.e., the target stroking stress), resulting in decreasing crush efficiency.

The samples with 1BI showed a different behavior, with the crush efficiency decreasing over the strain range of 0.1 to 0.3. The decrease corresponded to the second increase in the stress–strain curve. The sample showed a stepped behavior in its stress–strain curve, so the crush efficiency decreased during this time after reaching its maximum value of about 84% crush efficiency. The 1BI sample also showed a decrease in its crush efficiency from 0.3 strain onward. Once again, this corresponded with the plastic collapse region and then the densification region. Once the densification strain was reached, a monotonically decreasing linear behavior dominated the crush efficiency. At this point, the load was increasing, but the walls had collapsed, so that the sample could no longer transfer energy, so that crush efficiency was lost.

**Figure 9:**
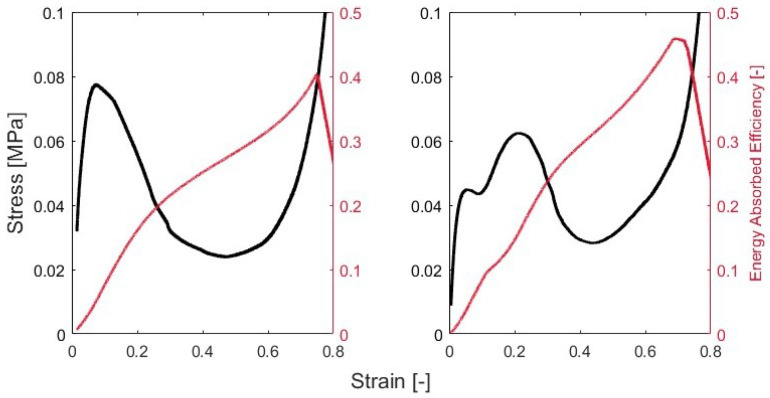
The stress–strain curves are plotted on the left axis with the energy-absorbed efficiency plotted on the right. The left-hand side shows the sample with no buckling initiators, and the graph on the right shows the sample with buckling initiators. The peak energy-absorbed efficiency strain corresponded with the threshold strain.

The second efficiency used to characterize the performance of the honeycomb was the energy-absorbed efficiency. The energy-absorbed efficiency monotonically increased until the peak stress was reached, at which point the EAE experienced a slight change in slope. As Figure 9 shows, the peak EAE occurred when the densification strain was reached for the 0BI samples. The maximum EAE for 0BI was 41% at a corresponding strain of 0.748. When reviewing the stress–strain curve for this quasi-static sample, the peak stress of 0.08 MPa was achieved for a second time at a threshold strain of 0.749. As validated by the knee in the EAE curve, the threshold strain was the same as the densification strain. These metrics are reported in Table 1.

For the 1BI samples, the same trend in EAE was identified. The EAE monotonically increased until the threshold strain of 0.729 was reached (Figure 9). At this strain, the peak stress of 0.064 MPa was reached for a second time. The maximum EAE for 1BI was 48% at a 0.729 strain, thus validating that the threshold strain was the same as the densification strain. For the quasi-static strain rates, the samples with 1BI outperformed the 0BI samples in terms of crush efficiency and energy absorption.

### 3.3. Low Strain-Rate Testing

Samples were also tested at two strain rates of 8.33 s${}^{-1}$ and 16.67 s${}^{-1}$, which are representative of low-velocity impacts (0.25 and 0.50 m/s respectively).

**Figure 10:**
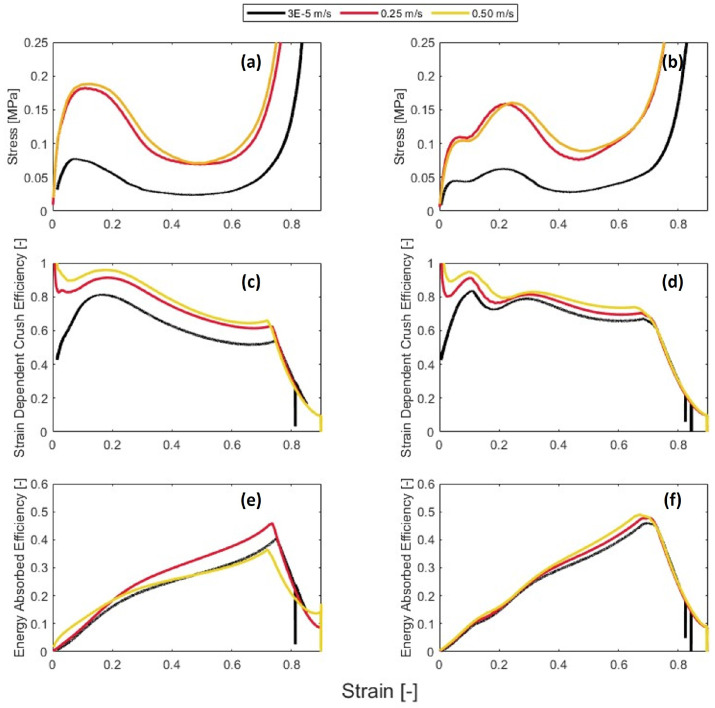
The behavior of visco-elastic honeycomb tested under three different strain rates. Left column shows no buckling initiators and right column shows samples with buckling initiators. (**a**,**b**) show stress-strain curves, (**c**,**d**) show crush efficiency, and (**e**,**f**) show energy absorbed efficiency.

The stress–strain curve for the 0BI samples tested at elevated strain rates showed similar characteristics to the quasi-static samples. The peak stress was shown to increase as the strain rate increased. The peak stress for the 0BI samples at 8.33 s${}^{-1}$ was 0.182 MPa, while the 16.67 s${}^{-1}$ peak stress was 0.189 MPa. This increasing peak stress can be seen in Figure 10a. The mean crush stress also increased with the strain rates. The mean crush stress for the 8.33 s${}^{-1}$ samples was 0.17 MPa, while the 16.67 s${}^{-1}$ stress was 0.18 MPa. Overall, the curves were shifted versions of the quasi-static samples. The stress–strain curves for the 1BI samples at low strain rates appeared to be shifted versions of the quasi-static samples as well. The peak stress increased from 0.159 MPa at a strain rate of 8.33 s${}^{-1}$ to 0.199 MPa at a strain rate of 16.67 s${}^{-1}$. This behavior also occurred with the mean crush stress. The mean crush stress for the 1BI samples tested at a strain rate of 8.33 s${}^{-1}$ was 0.13 MPa, and this increased to 0.16 MPa when the strain rate was increased to 16.67 s${}^{-1}$ (Figure 10b).

The crush efficiency shows that the 1BI samples outperformed the 0BI samples. When reviewing the crush efficiency data, there are two trends. The first is that the presence of buckling initiators does not degrade the crush efficiency as crush velocity increase. The 0BI samples averaged 91.34% and 96.02% at velocities of 0.25 m/s and 0.50 m/s, respectively. The 1BI samples averaged 91.46% and 93.86% at 0.25 m/s and 0.50 m/s, respectively. The samples crushed at a velocity of 0.25 m/s saw the crush efficiency maintained with the presence of buckling initiators, while the 0.50 m/s samples saw a slight decrease (Table 2). The second trend is that the crush efficiency increases as the strain rate increases. Together, these trends indicate that the 1BI sample was able to maintain its crush efficiency as strain rate was increased.

The energy absorption efficiency increased with an increase in the crush velocity. The 0BI samples had an average energy absorption efficiency of 46% at 0.25 m/s, which increased to 47% at 0.50 m/s. At higher velocities, the samples with buckling initiators outperformed their counterparts. The 1BI sample had an energy absorption efficiency of 49% at a strain rate of 0.25 m/s compared to an efficiency of 51% at a strain rate of 0.50 m/s.

### 3.4. Repeated Testing

As part of this study, the re-usability of the honeycomb was characterized. There were two purposes for these tests: to determine whether the samples could recover elastically after testing; and to quantify the effects on the energy absorption and crush efficiency of the samples.

The visco-elastic honeycomb was able to successfully rebound back to at least 98% of its original height with minimal defects. In reviewing Figure 6, both types of the samples successfully rebounded. The samples showed different folding patterns due to the presence or lack of buckling initiators, as discussed in Section 2.1.

**Figure 11:**
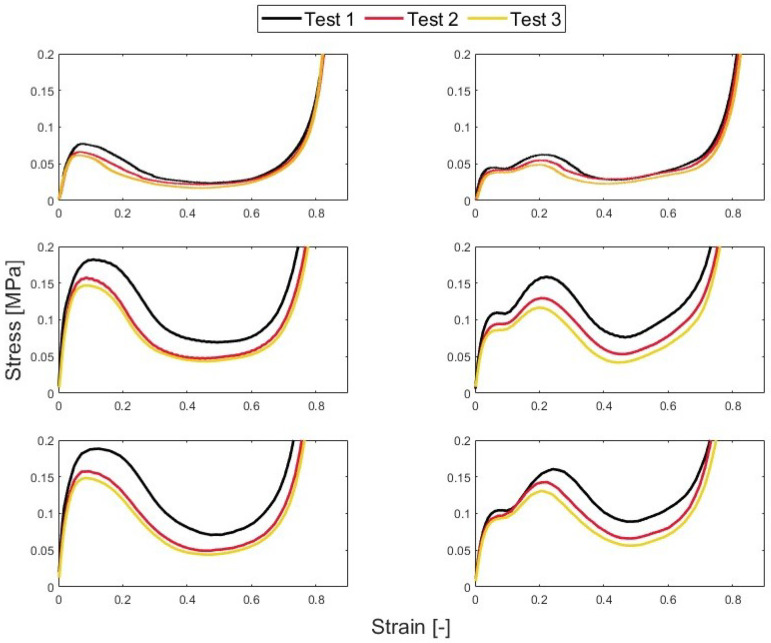
The stress–strain curves during three different tests. The left column is for 0BI and the right column is for 1BI. The top row is for the quasi-static conditions, the middle is for 0.25 m/s, and the bottom is for 0.50 m/s.

The re-usability was also analyzed by reviewing the performance metrics. As Figure 11 shows, the stress–strain curve shifted down with repeated testing. This makes sense because the sample had been pre-stressed. The honeycomb would be unable to support that much stress. The peak stress decreased from 0.081 MPa to 0.064 MPa for the 0BI samples that were tested in quasi-static conditions. When tested at an elevated strain rate of 16.67 s${}^{-1}$, the peak stress also decreased: from 0.189 MPa to 0.149 MPa. The samples with 1BI showed the same decreases in their peak stresses. In quasi-static conditions, the peak stress dropped by 0.013 MPa. Similarly, the 16.67 s${}^{-1}$ samples saw a decrease in the peak stress of 0.068 MPa (Table 3). Regardless of the strain rate, there was a decrease in the peak stress over continued testing cycles.

Although the crush efficiency showed the same trend, it was less drastic of a decrease. The samples with 0BI that were tested at quasi-static strain rates showed that their crush efficiency decreased by 0.43% from the first test to the third. The 1BI samples showed a 4.16% decrease. Implementing the buckling initiators yielded a similar decrease in the crush efficiency with repeated testing. The samples that were tested at 0.25 m/s showed a 1.9% and 0.70% decrease for the 0BI and 1BI samples, respectively. Similarly, the samples tested at 0.50 m/s showed a 1.49% and 0.64% decrease. As the crush velocity increased, the buckling initiator samples showed a smaller decrease in their crush efficiency after multiple tests.

As anticipated, there was some degradation in the honeycombs, but these showed an ability to rebound and were able to be used for additional tests. The presence of buckling initiators minimized the degree of damage in the honeycomb structure.

## 4. Analysis

A finite element analysis was conducted in ABAQUS to simulate the crush efficiencyof the honeycomb at a strain rate of 3 × $10^{-5}$ (or quasi-static conditions). The simulation was intended to better understand the influence of the buckling initiators on the honeycomb. The model selected was the imperfect perturbation factor, which was chosen so that the buckling modes could be properly captured. The elastic material was modeled using a Young’s modulus of 40 MPa and a density of 1053 kg/m^3^.

The samples with 1BI were modeled using a standard surface-to-surface contact with a friction factor of 0.9 at the bottom interface and 0.6 at the upper interface. These values were selected because the FEA models aligned closest to the experimental behavior of the honeycomb. The linear buckling mode shape perturbation scale factors were 0.085 for modes 1 to 8. To model the wall thickness at the buckling initiator, a smaller wall thickness was used. The honeycomb had a modeled walled thickness of 0.85 mm except for in the buckling initiator region. At this location, the wall thickness was identified as 0.65 mm. To model the behavior of the honeycomb, at least one degree of freedom was required. The friction factor was identified to be this degree of freedom. The other factors in the study were set and the friction factors were tuned until an optimal stress–strain curve was achieved.

**Figure 12:**
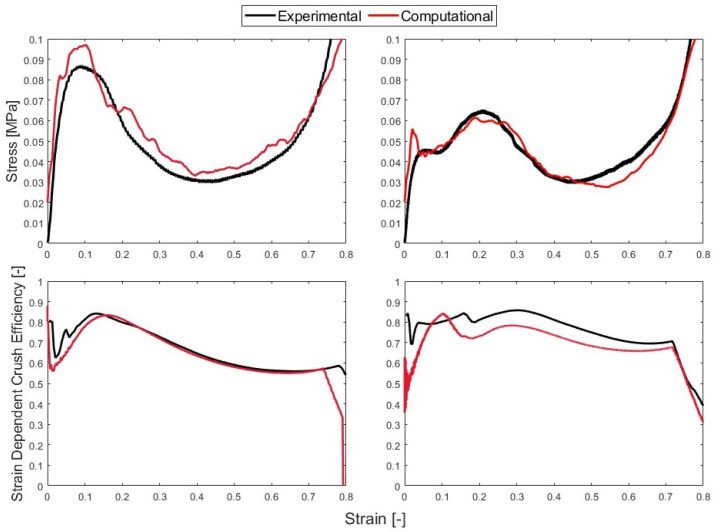
The computational and experimental stress–strain curves for the samples tested at a quasi-static strain rate in the top row and the strain-dependent crush efficiency in the bottom row. The left column shows the 0BI samples and the right column shows the 1BI samples.

The performance of the 1BI quasi-static honeycomb showed a good agreement between the model and the experimental results, as shown in Figure 12. The model shows a crush efficiency of 85% compared to the experimental results of 84%.

Using similar conditions, the quasi-static 0BI can be modeled. For the 0BI sample, the simulation used a standard surface-to-surface contact with a friction factor of 0.01 early in the experiment and near the end, and this was value was used due to the honeycomb losing contact with the platens. As the sample densified, the friction factor used was 0.9 at the bottom interface and 0.6 at the upper interface. This was due to the honeycomb maintaining direct contact with the platens during that time. Because there were no buckling initiators, the wall thickness was 0.85 mm. The samples with 0BI required two sets of friction factors to match the experimental curves. It was observed in the experiment videos that the top and bottom edges of the honeycomb samples initially had partial contact with the platens, but that this contact off th edges improved greatly as loading increased.

The model for the 0BI samples slightly overpredicted the crush efficiency. The model showed a projected crush efficiency of 84%, while the experimental results showed a crush efficiency of 82%. The behavior of the stress–strain curve followed the experimental performance of the honeycomb, as shown in Figure 12. The behavior computational model was a near perfect prediction of the 0BI strain-dependent crush efficiency; however, it under predicted the 1BI performance over the full strain range. In the low-strain range (less than 0.1 strain), there was a difference between the computational and experimental results regardless of the sample analyzed, as Figure 12 shows. The honeycomb did not see this low of a strain, so it can be ignored. As the strain increased, the 0BI samples showed good agreement between the experimental and computational model; however, the 1BI model under predicted the crush efficiency across the full strain range.

When reviewing all metrics, the computational analysis was able to represent the performance of the experimental data at quasi-static strain rates. A full summary of the performance of the computational data is provided in Table 4. The peak stress and crush efficiency were accurately represented with the computational model, which validates the experimental work conducted.

## 5. Discussion

**Figure 13:**
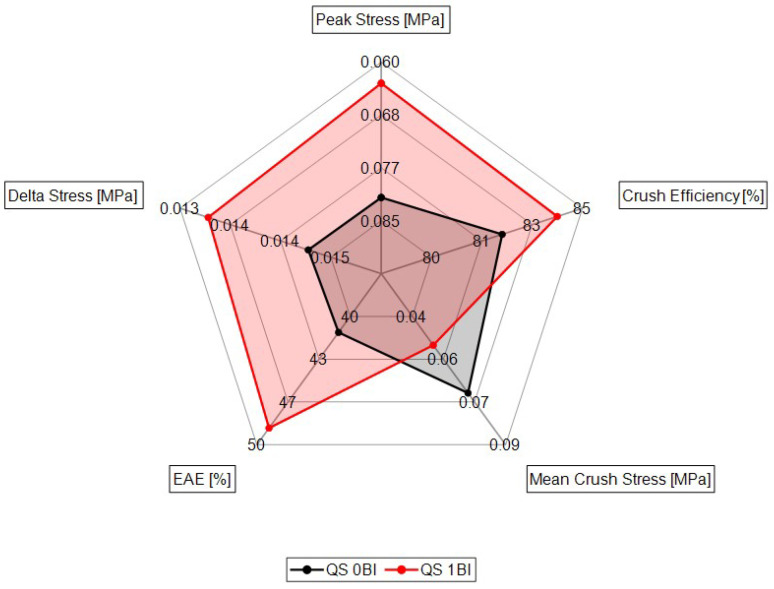
The radar plot shows the performance of the quasi-static conditions with the 0BI and 1BI samples in regard to these particular metrics.

Throughout this study, five metrics were used to compare the performance of the samples with and without buckling initiators in a variety of strain rates. To assist in understanding which type of samples were most effective, a radar plot was used. Around the exterior of the plot are the metrics, as Figure 13 shows. The axis for each metric was oriented so that the axis increases or decreases depending on which denotes the best performance. If one sample encompasses another, it denotes the best solution (as defined by the metrics).

When reviewing the quasi-static results, the 1BI samples encompassed the 0BI sample, indicating the IBI samples have the best geometry given these metrics. The 1BI samples showed a lower peak stress and higher change in stress value, indicating a higher crush efficiency. All three of these metrics were dominated by the 1BI samples, along with the energy absorbed efficiency. These samples were also more efficient at transferring crush energy.

## 6. Conclusions

Visco-elastic TPE honeycomb manufactured with 1BI have been shown to outperform their 0BI counterparts with regard to the metrics set forth by this study. The following conclusions can be drawn from this work.

1. Visco-elastic honeycomb with buckling initiators experiences axisymmetric failure as opposed to their counterparts that are without buckling initiators. Folding initiates at the buckling initiator and continues until the sample has been folded in a controlled manner. The samples without buckling initiators experience shear buckling, which results in non-axisymmetric folding patterns.
2. Visco-elastic honeycomb is able to rebound to over 95% of its initial height after testing three different periods. The honeycomb experiences some permanent deformation, but following 24 h, it is able to be reused.
3. Visco-elastic honeycomb with buckling initiators that are located midway up the sample are more crashworthy in quasi-static applications. The buckling initiators assist in lowering the peak stress, thus increasing the strain-dependent crush efficiency. Similarly, the energy-absorbed efficiency is shown to increase with the presence of buckling initiators.
4. Visco-elastic honeycomb with buckling initiators increases the energy-absorbed efficiency and decreases the peak stress when implemented in low-strain rate settings. The crush efficiency is preserved as the strain increases when buckling initiators are implemented.
5. After repeated testing at low-strain rates, the visco-elastic honeycomb with buckling initiators experiences less degradation in its crush performance. Thus, these visco-elastic honeycomb structures were observed to provide deformation recovery when using this type of buckling initiator. Protection against multiple impacts can be provided, albeit with reduced effectiveness.
6. Numerical analysis validated the experimental results for samples with and without buckling initiators in quasi-static conditions.

In the future, the behavior of these samples should be characterized at high-strain rates. The low-strain rate environment has been characterized; however, most impact and energy absorption events for humans begin at 6.7 m/s. Quantifying the behavior of these energy absorbers at higher velocities will further define the performance over a higher range of safety applications.

## Figures and Tables

**Table 1:** The average properties for the quasi-static strain rate samples.

BI	$\sigma_{\mathrm{pk}}$	$\sigma_{\mathrm{mc}}$	Δσ	$\eta_{\mathrm{SD}}$	$\eta_{\mathrm{EA}}$	${\epsilon |}_{\eta_{\mathrm{EA}}}$
	[MPa]	[MPa]	[MPa]	[%]	[%]	[-]
0	0.08	0.07	0.014	82.07	41.24	0.749
1	0.06	0.05	0.013	84.08	48.70	0.711

**Table 2:** The average properties for the low speed samples.

V	BI	$\sigma_{\mathrm{pk}}$	$\sigma_{\mathrm{mc}}$	Δσ	$\eta_{\mathrm{SD}}$	$\eta_{\mathrm{EA}}$	${\epsilon |}_{\eta_{\mathrm{EA}}}$
[m/s]		[MPa]	[MPa]	[MPa]	[%]	[%]	[-]
0.25	0	0.18	0.167	0.016	91.34	45.97	0.733
0.25	1	0.16	0.130	0.030	91.46	49.73	0.702
0.50	0	0.19	0.181	0.008	96.02	47.49	0.720
0.50	1	0.20	0.161	0.038	93.86	51.72	0.706

**Table 3:** The decrease in the metrics when comparing the following tests to the first test.

V	BI	Test	$\sigma_{\mathrm{pk}}$	$\sigma_{\mathrm{mc}}$	Δσ	$\eta_{\mathrm{SD}}$	$\eta_{\mathrm{EA}}$	${\epsilon |}_{\eta_{\mathrm{EA}}}$
m/s			[MPa]	[MPa]	[MPa]	[%]	[%]	[-]
0.25	0	2	0.157	0.140	0.017	89.18	41.14	0.748
0.25	0	3	0.148	0.132	0.015	89.62	41.08	0.751
0.25	1	2	0.130	0.107	0.023	90.17	48.20	0.708
0.25	1	3	0.117	0.097	0.020	90.76	46.58	0.708
0.50	0	2	0.158	0.150	0.008	95.14	43.88	0.735
0.50	0	3	0.149	0.141	0.008	94.53	42.50	0.735
0.50	1	2	0.143	0.119	0.024	94.42	48.51	0.699
0.50	1	3	0.131	0.110	0.021	93.22	48.41	0.706

**Table 4:** The properties of the experimental and computational quasi-static samples.

	$\sigma_{\mathrm{pk}}$			$\eta_{\mathrm{SD}}$			$\sigma_{\mathrm{mc}}$		
	[MPa]			[%]			[MPa]		
BI	Exp	Comp	% Diff	Exp	Comp	% Diff	Exp	Comp	% Diff
0	0.086	0.096	10.4	82.07	84.13	2.48	0.07	0.08	13.3
1	0.065	0.061	6.15	84.08	85.73	1.94	0.06	0.05	18.1

## Data Availability

Data available upon reasonable request.
